# Supplementary material for: Are carbon emissions trading and green financial instruments synergistic? -Comprehensive quantitative research based on content analysis
Source: PLoS One. 2024 Mar 7;19(3):e0298601. doi: 10.1371/journal.pone.0298601 (PMC10919601; doi:10.1371/journal.pone.0298601)
Supplement: S1 Data — (ZIP) [file pone.0298601.s001.zip › Data/地级市Z_F工作报告.zip!/╔┬╬≈/▒a╝a╩╨/└┤╫╘╣1⁄2╓┌║┼í╛┬φ┐╦╩2╛▌═°í┐_2014.docx]

各位代表：

　　现在，我代表市人民政府向大会作工作报告，请予审议，并请市政协委员和其他列席人员提出意见。

2013年工作回顾

　　过去的一年，在市委的坚强领导下，市政府紧紧围绕“打造关天副中心、建设和谐新宝鸡，实现六个走在前列”的奋斗目标，聚焦发展不放松，科学施政求突破，攻坚克难，创新实干，圆满完成了市十四届人大三次会议确定的目标任务。全年实现地区生产总值1545.91亿元，比上年增长13%，高出全省增速2个百分点；地方财政收入72.02亿元，增长16.4%；城乡居民人均收入分别达到28509元、8376元，增长10.6%和13.6%。

　　主导产业快速增长。面对诸多不利因素，迎难而上调结构，抓住机遇促转型，经济实力持续增强。工业规模不断扩张。新增规模以上工业企业64户，规模以上工业实现增加值744.67亿元，12户大企业大集团工业总产值突破千亿，宝鸡卷烟厂销售收入过百亿，东岭集团在中国企业500强中上升89位，“宝钛”、“凌云”商标被认定为中国驰名商标。实施了百户配套企业成长计划和中小企业成长工程，新增中小企业3567户。高新区被确定为全国首批专利导航产业发展实验区，蔡家坡经开区晋升国家级经开区工作取得实质性进展，11个省级重点县域工业园区实现总产值772亿元，增长30.6%。省电子信息集团重组渭河工模具总厂全面完成，宝鸡叉车五厂等企业实现了转型脱困。新创建国家级企业技术中心1个、省级企业技术中心6个，全省首家地市级科技创新服务中心建成投用，被命名为全国科技进步考核先进市、国家科技成果转化服务示范基地、陕西省钛及钛合金科技产业示范基地。现代农业步伐加快。粮食总产达到145.59万吨，扶风县成为国家级小麦良种繁育标准化示范区。奶牛存栏继续位居全省前列。新增苹果、猕猴桃、核桃等经济林27.1万亩，千阳县成为全国最大的矮砧集约苹果生产基地，陇县荣获中国核桃之乡称号。蔬菜种植面积达到76.81万亩，太白贝母跻身国家地理标志产品保护行列。新发展市级农业产业化重点龙头企业40户，农民专业合作社293个。现代农业发展创出新模式，新建省级现代农业园区10个、市级35个，眉县猕猴桃产业园区荣获“国家农业产业化示范基地”，凤县坪木镇被评为全国一村一品示范乡镇。全国农社对接现场会在我市召开，陈仓区绿丰源产供销一体化新模式在全国推广。完成231个行政村电网升级改造，新建基本农田7.8万亩，发展节水灌溉13万亩，治理水土流失400平方公里，解决了14.1万农村人口饮水安全问题。第三产业蓬勃发展。太白山景区改造升级基本完成，通天河森林公园跻身国家4A级旅游景区，茵香河文化旅游区被命名为中国文化产业示范区，中央电视台投资的《英雄时代•炎黄大帝》电视剧在宝鸡完成拍摄，法门寺佛文化景区、岐山西周文化景区进入全省十大文化旅游景区，凤县、眉县跻身中国旅游百强县，宝鸡荣获亚洲旅游业金旅奖。全年接待国内外游客3625万人次，实现旅游综合收入236.4亿元，分别增长32%和34%，位居全省前列。全年新增限额以上商贸流通企业222户。外贸进出口逆势增长，总额位居全省第二。成功举办了首届宝鸡金融博览会，新引进光大银行、海通证券等6户金融机构，秦川发展、东岭集团企业债获批发行，陕西高端装备制造创投基金挂牌成立，全年新增贷款125.41亿元，金融业规模和实力进一步提升。

　　重大项目加快推进。重点项目数、投资额、开工率、招商引资到位资金等均创历年之最，为宝鸡发展奠定了坚实基础。300个市级重点项目完成投资546.97亿元，完成率106.18%。重大产业项目建设成绩斐然，农夫山泉一期、长青能化甲醇一期、郭家河煤矿等81个项目建成投用，青岛啤酒宝鸡工业园一期、海螺型材等项目加快推进，国铁机车基地等项目开工建设。交通枢纽地位进一步巩固，3条高速、5条国道进入国家公路网规划，西宝客专建成通车，西宝北线改造全面完成，连霍高速市区过境段、绛法高速、西宝中线陈仓段改扩建等项目进展顺利，宝鸡支线机场前期工作取得突破性进展。完成全社会固定资产投资1669.78亿元，其中民间投资达到907.66亿元。招商引资工作亮点纷呈，引进了吉利汽车、西部光谷光通信产业园等一批重大项目，全年落实招商引资项目871个，到位资金648.33亿元，居全省前列。争取中省资金150.4亿元，创历史新高。

　　新型城镇化加力提速。城市新区建设步伐加快，蟠龙新区龙盘西路基本建成，南客站片区拆迁改造全面铺开，高新大道三期全线建成通车。城市基础设施更加完善，北新路一期、滨河北路等小道路完成改造，体育场周边、川陕路等道路排水工程建成投用，新增供热面积200万平方米，市域天然气环网西环部分基本完工。新东岭、华东万润城、虢镇东门十字等城市综合体项目加速推进。长青路立体停车场、公共自行车系统一期建成投用，新增公交车101辆。城镇载体服务功能明显提升，3个市域副中心、7个县城扩容提质，新增建成区面积6平方公里。4个省级重点示范镇完成投资22.28亿元，陇县固关镇列入全国少数民族特色村寨保护项目，太白县黄柏塬镇荣获“最向往的美丽中国特色镇”称号。13.45万农民进城落户，全市城镇化率预计达到47.41%，比上年提高1.8个百分点。

　　生态优势日益彰显。渭河综合治理继续走在全省前列，完成堤顶路面硬化65.6公里，建成绿色生态景观长廊70公里。农村环境综合整治经验在全省推广，88个村整治任务高标准完成。拆除燃煤锅炉210台，市区空气质量二级及以上天数达到316天。绿色宝鸡建设成效明显，完成造林46万亩，市区及城镇新增绿地3.5万亩。太白、麟游荣获全国绿化模范县，扶风、陇县荣获国家园林县城称号。国家生态市创建深入开展，凤县国家级生态县通过评估，全市累计建成国家级生态镇19个。

　　民生保障更加有力。民生十大领域完成投资80.2亿元，居民幸福指数位列全省第二。城镇新增就业5.8万人，农村劳动力转移就业105.6万人。大病保险试点启动实施，城乡居民养老保险并轨走在西部前列。保障性住房建设位居全省第一，新开工3.72万套，建成2.93万套，累计分配入住6.56万套。教育强县占到全省五分之一，太白县创建义务教育发展基本均衡县通过国家验收，扶风县荣获全国农村义务教育学生营养改善计划先进县。新建改建幼儿园35所，实现乡镇公办中心幼儿园全覆盖，完成245个薄弱学校改造项目，市特殊教育学校和一批数字化校园项目建成投用。全国国家卫生城市创建现场会在我市召开，被评为全国卫生城市创建典型市。在全省率先对失独家庭发放补助资金1102万元。成功创建为首批国家公共文化服务体系示范区，新建省级文化产业示范基地5个，激情广场荣获第十届中国艺术节群星奖，举办了中国首届社火艺术节，陇县地台社火、陇州花灯荣获中国民间艺术山花奖。10处遗址列入全国重点文物保护单位，九成宫和北首岭遗址保护规划通过省政府颁布，石鼓山考古发掘再一次引起轰动。承办了五项全国大型体育比赛，省十五运会筹备工作有序推进，全国全民健身示范城市各项指标全面完成，麟游、太白荣获全国群众体育先进县称号。渭滨区建成全市首个居家养老服务信息平台,全市新建村级老年活动中心449个。率先在西部开展残疾预防综合试点，投入8800万元为残疾人办了十件实事。建设扶贫整村推进项目村89个、“三无户”安全住房1000户，11.8万农村贫困人口实现脱贫。

　　社会管理不断创新。社会治安满意率、平安创建知晓率、政法队伍满意度均列全省第一，连续三年被评为全省社会管理综合治理工作优秀市。全国节水型社会建设试点通过国家验收，被列为国家智慧城市试点。再次蝉联全国双拥模范城市称号，成为全国社会组织建设创新示范区。社区网格化管理得到强化。荣获全国法治城市创建活动先进单位，麟游县被评为全国法治县创建先进县。食品药品安全示范县区创建深入推进，金台区荣获全国首批餐饮服务食品安全示范区。渭滨区荣获全国县级防震减灾工作综合考核先进单位。深化“打非治违”专项行动，安全生产形势持续好转。高度重视人大代表建议、政协委员提案办理工作，共办理建议、提案947件，办结率100%。全面清理市级行政职权，扎实推进九大领域政府信息公开，开通了市政府官方微博，开展了邀请公民代表走进市政府活动，创办的“百姓问政”电视直播节目在全省推广，干部作风明显改进，为民服务水平进一步提高。同时，监察、审计、国土、统计、工商、质监、人防、物价、气象、档案、地震、地志、民族宗教、国家安全、妇女儿童、侨务外事、红十字会等工作也取得了新的成绩。

　　各位代表，过去一年，是我市经济实力持续提升、发展后劲显著增强的一年，是城乡面貌明显改善、人民群众得到更多实惠的一年，是各项工作亮点频显、对外知名度和影响力大幅提升的一年。这些成绩的取得，是市委总揽全局、正确领导的结果，是市人大、市政协监督支持、共谋发展的结果，是全市人民同心同德、顽强拼搏的结果。在此，我代表市政府，向人大代表、政协委员、各民主党派、各人民团体、社会各界人士，向驻宝企事业单位、人民解放军、武警驻宝部队全体官兵、公安干警，表示崇高的敬意和诚挚的感谢！

　　在充分肯定成绩的同时，我们也要正视发展中面临的困难和问题，主要是：经济总量不大，发展方式有待进一步转变；第三产业发展滞后，旅游业缺乏龙头景区，非公经济发展不快，县域经济实力不强；城镇化水平还不高，城镇化率低于全国、全省平均水平；政府职能转变还不到位，一些干部的思想作风还不能适应新形势、新任务的需要。对于这些问题，我们将高度重视，采取有力措施，切实加以解决，绝不辜负全市人民的期望和重托！

2014年政府工作

　　今年，是全面深化改革的起步之年，也是我市实现提质增效、加快发展的关键一年。当前，世界金融危机影响仍未消除，国际市场竞争更趋激烈，世界经济缓慢复苏态势仍将延续。但我国经济发展长期向好的基本面没有改变，国家将继续保持宏观经济政策的连续性和稳定性。同时，《全国老工业基地调整改造规划》深入实施，《国家新型城镇化规划》即将颁布，丝绸之路经济带正在规划建设，向西开放进一步扩大，这些都为宝鸡发展带来了难得机遇。我们必须坚持底线思维，增强忧患意识，抢抓机遇，乘势而上，奋力开创宝鸡改革发展新局面。

　　2014年政府工作的总体要求是：认真贯彻落实党的十八大、十八届三中全会和省、市委全会精神，以全面深化改革为主线，以稳中求进为总基调，突出做大总量、做优质量两大要务，在转型升级中提质增效，在改革开放中激发活力，在转变职能中增添动力，在改善民生中促进和谐，加快打造关天副中心、建设和谐新宝鸡，努力走在建设“三个陕西”的前列。

　　主要预期目标是：生产总值增速高于全省两个百分点、达到13%左右，全社会固定资产投资增长26%，社会消费品零售总额增长14%，地方财政收入同口径增长13%，规模以上工业增加值增长16%以上，城镇登记失业率控制在4%以内，城乡居民人均收入分别增长12%、13%，人口自然增长率控制在5.5‰以内，万元GDP能耗下降3.5%。

　　重点抓好八个方面工作：

　　一、突出特色优势产业，推动工业转型升级

聚力“四大主战场”。“宝鸡•中国钛谷”、吉利汽车项目、西凤酒城建设、麟北煤田开发，投资规模大、产业关联度高，是我市长远发展的坚实基础和重要支撑，是经济发展的生力军，必须全力攻坚、重点突破，使其做大做强、迅速形成规模。编制完成并启动实施钛谷发展规划和东区（眉县）建设规划，加快国家钛材产品质量监督检验中心、钛谷有色金属交易中心、陈仓道北新区钛科新城等项目建设。支持宝钛集团发挥龙头企业优势，加强行业内企业间深度合作，提升产业整体水平。全力推进吉利汽车项目建设，切实做好协调服务和环境保障工作，确保项目周边道路按期建成。围绕吉利汽车项目配套，引导更多企业进入配套体系，延长产业链条。加快推进西凤酒城建设，加大西凤大道和酒城供热管网、污水处理厂等基础设施建设力度，确保签约配套项目落地开工。加速推进麟北煤田开发，密切与中省有关部门的沟通衔接，力争《永陇矿区麟游区总体规划（修编）》尽快获得国家批复。加快招贤、园子沟煤矿相关工作进度，全市煤炭产量突破1000万吨。抓好宝麟铁路建设，确保年内竣工通车。坚持开发与生态保护并重，加大煤炭综合利用力度，推动煤炭产业可持续发展。

　　壮大优势工业。围绕九大产业集群，推动传统工业稳定增长、创新突破，提升产业竞争力。加快高新区汽车零配件创业孵化基地、通力专用车生产线等项目建设，力争凌云蓄电池、新利达汽车零部件生产线等项目建成投产，壮大汽车及零部件产业集群。加快高端装备石油产业园、宝鸡机床集团配套园、航天航空产业基地等建设，提高工艺技术水平，着力打造高端装备制造业基地，年内规模以上装备制造业产值达到600亿元。切实强化保障服务，加快实施阜丰生物搬迁，确保青岛啤酒宝鸡工业园一期、今麦郎面粉挂面生产线、农夫山泉二期建成投产，促进烟酒食品产业快速发展。做好长青能化铁路支线建设前期工作，积极实施宝氮集团搬迁技改等项目，年内能源化工产业实现产值280亿元。

　　培育新兴产业。围绕新能源、新材料、新医药、新一代信息技术产业等重点领域，以总部经济、网络经济和创新经济为重点，加强规划引导，加大扶持力度，引龙头、强配套、促聚集，加速新兴产业集群化、规模化发展。大力推进国家信息消费试点市建设，制定出台互联网产业发展规划，支持渭滨区建设移动互联网产业园。加快西部光谷光通信产业园一期、千阳环保新材料、陇县关山风电、凤县风电、太白龙源风电等项目建设，确保年内建成投用。

　　加快县域工业发展。把工业园区作为县域经济发展的重要载体，按照“高起点规划、高标准建设、高效率推进”的思路，下功夫完善工业园区基础设施，提前做好土地征用、规划报批、标准化厂房建设等工作，着力提升园区承载能力，确保项目引得来、落得下、建得快、长得大。全市17个省级重点县域工业园区总产值力争突破850亿元。启动重点县区中小企业服务平台建设，用好1000万元小微企业发展基金，抓好企业家培育和中小企业成长工程，年内发展规模以上工业企业40户以上。充分发挥市担保公司作用，推动县区设立中小企业信用担保机构，有效缓解非公企业融资难题。

提升工业创新能力。支持秦川集团争创国家数控磨床工程技术研究中心，抓好高速铁路工程机械等省市级工程技术研究中心、企业技术中心建设，新培育省、市级创新型（试点）企业10户以上。加快高新区创新型科技园区、国家知识产权示范园区、钛产业专利导航发展实验区建设步伐，加大钛材料、石油装备、航空安全等领域孵化器、加速器建设力度，筹建低碳钛材及产品标准化委员会，提升创新实力。健全技术创新市场导向机制、产学研协同创新机制和效益评价机制，强化企业技术创新的主体地位，激发创新活力。加强与北大、清华等院校的校地、校企合作，为加快发展提供人才和智力支撑。

　　二、加大项目建设力度，强化投资拉动作用

　　积极谋划争取项目。紧跟国家产业政策和投资方向，针对性地谋划一批增后劲、管长远、带全局的重大项目。围绕新兴产业发展、重大产业项目配套和大企业大集团扩张，谋划包装一批衍生项目。加强对中省政策的研究，策划包装一批基础设施、产业转型升级和民生保障等方面的项目，主动对接，争取更多的项目列入中、省计划盘子。全年争取中省资金150亿元以上。

　　全力抓好招商引资。瞄准国内外知名企业、著名品牌进行招商，力争引进世界500强或中国500强企业2户以上。支持在宝外埠商会快速发展，在发达地区扶持发展一批宝鸡商会，发挥商会和招商顾问的桥梁纽带作用，大力开展以会招商、以商招商。积极参加西洽会、陕粤港澳经济合作周等活动，全力抓好项目落地。总结吉利项目落户经验，主动出击，跟踪服务。全年招商引资到位资金增长20%以上。

　　强力推动项目建设。今年，全市安排市级重点项目340个，年度投资590亿元以上。围绕项目建设，通过争取用地指标、盘活土地存量、集约节约利用等措施，解决用地问题。积极争取中省投资，加大信贷投入，聚拢民间资金，解决投资问题。精简审批事项，优化服务流程，缩短土地利用、规划建设、节能审查、环境影响评价等环节工作周期，做好环境保障，最大限度提高效率。

　　努力壮大财源规模。充分发挥财税政策的导向作用，强化基础财源、壮大主导财源、培育新兴财源、发展后备财源。坚持“引资”与“引税”并重，大力引进市场前景好、经济效益高、环境污染小、财税贡献大的项目，优化产业结构，提高产业化项目占比，增强创新创税能力，加快形成新的骨干财源。大力发展第三产业和非公经济，促进财源结构多元化。研究制定财政激励考核新办法，调动各级增加收入的积极性。严格控制和规范政府举债、担保行为，着力防控债务风险。

　　三、大力发展现代农业，促进农村全面繁荣

　　扩张特色农业规模。毫不放松抓好粮食生产，守住耕地红线，调整生产结构，推广良种良法，加大补贴力度，确保总产达到145万吨以上，保障粮食安全。大力推进规模化养殖和集约化经营，建设全国高产奶牛养殖和高端乳制品加工基地。推广秦宝肉牛模式，发展肉羊养殖大场大户，建设百万头肉牛肉羊产业基地。加快建设渭北塬区苹果产业带，新发展苹果11万亩。在岐山、扶风、凤翔等县区，大力推广千阳“海升”模式，打造全国最大的中早熟矮化苹果基地。以眉县为核心，辐射带动秦岭北麓新发展猕猴桃3万亩，建设全国最大的优质猕猴桃基地。以太白煜群、千阳锦泰为龙头，建设绿色无公害蔬菜基地。以陈仓区绿丰源蔬菜基地为样板，在川塬县区集中连片建造一批千亩蔬菜园，建设设施蔬菜基地。以秦岭山区、关山、乔山为重点，新发展核桃、花椒等干杂果10万亩，建设干杂果产业基地，推动特色产业蓬勃发展。

　　创新农业发展模式。做大做优扶风—眉县国家级休闲农业示范区和25个省级现代农业园区，积极争创陈仓国家级现代农业示范区，新增省级园区3个、市级园区30个，引领全市农业向规模化、集约化、专业化、标准化迈进。培育新型农民，提升技能素质。全力抓好眉县国家级猕猴桃批发交易中心和陕甘宁国际农产品物流中心建设，培育壮大龙头企业，发展农产品加工园区，建设农产品物流配送及深加工中心，有效提升农业综合效益，带动农民增收致富。

　　持续改善农村条件。加大农田水利设施建设力度，恢复改善灌溉面积10万亩，发展节水灌溉2万亩，建设眉县金渠规模化高效节水灌溉示范区。实施小流域综合治理，新修改造基本农田4万亩，治理中小河流4条，治理水土流失面积200平方公里，积极创建省级水土保持示范园，持续改善水生态环境。推进农村安全饮水工程建设，解决10万农村人口安全饮水问题。加快千河流域防洪治理、麟游普化水库、千阳冯坊河水库等工程前期工作，增强农业可持续发展能力。完成农村电网改造5375户，新建供电线路111千米，全面改善农村生产生活条件。

　　四、加快发展服务业，提高三产贡献份额

　　打造一流旅游目的地。我市旅游资源丰富，发展文化旅游产业得天独厚。抢抓西宝客专开通机遇，面向国内外加大推介力度，积极引进有实力、有能力的企业集团，在资金投入、策划包装、运营管理等方面实现新突破，努力打造在全国有较大影响的旅游景点。编制完成全市工业旅游、都市旅游、乡村旅游、秦岭北麓片区旅游发展总体规划和建设性详规。抓好法门寺佛文化景区建设，办好世界佛教徒联谊会第27届大会，确保年内创建为国家5A级景区。推进太白山国家级文化产业示范区创建，力争创建成国家5A级景区。加速茵香河区域开发，实施金台观二期建设，打造都市旅游区。高标准编制岐山西周文化景区规划，启动实施凤凰山遗址公园、中华周文化城、周原国际考古研究基地等项目建设。强力推进关山草原、黄柏塬、凤县古羌文化旅游产业示范区、灵山先秦古镇生态文化产业园等景区标准化建设，打造旅游新亮点，年内力争创建国家4A级景区6家、3A级景区8家。面向全国开展旅游宣传及项目招商，全面推介七大主题精品线路，推进智慧旅游城市建设，办好宝鸡文化旅游节，扩大宝鸡美誉度和影响力。全年旅游综合收入增长30%以上。

　　加快建设西部商贸物流中心。引进高端商贸企业，推进商贸设施建设，优化完善网点布局。加快海航国际广场、石鼓文化城太阳市商业街等项目建设进度，确保红星美凯龙、世纪荟萃广场、巨一时代广场等建成投用。加快新东岭等城市综合体建设步伐，开工建设银泰商务中心和皇冠假日酒店。加快商贸流通体系建设，继续推进万村千乡市场、农超对接、平价商店等工程，方便群众生活。加快宝鸡再生资源产业园建设，实现再生资源综合利用。开工建设陈仓物流园区物流信息大厦，加快六星（宝鸡）国际汽车生态文化产业园等项目进度，确保眉县煤炭物流园、招贤物流中心建成投用。完善城市夜间购物、餐饮、文化娱乐、旅游休闲等功能，发掘一批夜间消费产品和活动项目，促进和扩大消费。

　　努力扩大对外贸易。加快向西开放步伐，打造丝绸之路经济带新的战略枢纽。推进宝鸡综合保税区各项工作，引进一批支撑性企业，搭建开放新平台。强力实施外贸振兴“四大工程”，抓好25户龙头企业培育，支持宝钛等10户重点企业扩大进出口。继续加大对专业性外贸公司的政策、资金扶持力度，促进出口供货转化。依托外贸孵化中心，力争全年孵化实绩企业20户，培育新的外贸增长点，实现对外贸易进出口总值9.77亿美元。

　　提高金融服务实体经济能力。以建立多元化、多层次的金融组织体系为目标，鼓励辖区内银行在县城和工业园区增设网点，扩大覆盖面。支持符合条件的民营资本参与农信社增资扩股、设立中小银行等金融机构，加快眉县、千阳、麟游、凤县信用联社改制商业银行步伐，推进“政府补贴贷”等金融产品创新。支持优势企业发行企业债、公司债，推动中小企业在新三板上市。全年新增贷款110亿元以上，社会融资规模160亿元以上。

　　五、推进新型城镇化进程，提升辐射带动能力

　　高起点规划城镇布局。结合关天副中心城市建设，依托宝鸡独特的山水脉络、历史文化，修订、完善和优化城镇布局规划。强化城市设计，对主城区、渭河两岸单体建筑合理控制，把握建筑色彩、立面平面布局、周边环境等要素，形成高低错落有致、绿地空间合理、建筑形式新颖的现代城建格局，构建城市建筑的旋律美、整体美、和谐美。大力推广生态建筑、节能环保建筑，提高绿色建筑在新建建筑中的比重。强化规划管理，保证规划的严肃性和连续性，引领城市科学发展。

　　高品位打造城市新区。全面推进蟠龙新区开发建设，加快龙腾路、旭东路等七条道路及地下管网工程建设，确保蟠龙大道、龙盘西路及供水工程建成投用，开工建设蟠龙公园。加速南客站片区开发，开工建设站南大道，确保站前广场、站前大道年内建成投用。加大陈仓区基础设施建设力度，开工建设北新路二期，加快东冠世纪城项目进度，完善城市载体功能。加快高新区建设步伐，全线贯通高新1—30路，完成科技新城“两横四纵”六条道路和配套管网建设。

　　高标准提升主城区功能。按照“完善功能、提升形象”的思路，加快老城区服务功能完善。抓好宝光路、北环路、川陕路东段改造，年内建成金陵三桥、金陵西路、龙山路排水工程。大力推广代家湾城中村改造模式，加快三合村、光明村等城中村改造步伐。实施天然气三期利用工程，新发展用户2万户。加快群众路供热管网、陈仓区集中供热入户工程，新增入网面积100万平方米。新建一批市场、停车场、公厕等设施，调整优化公交线路，延长运营时间，方便群众生活。

　　高质量建设城镇群。制定出台政策措施，合力推动三个市域副中心加快发展，完善蔡家坡城区道路、供排水、供热等基础设施，加大教育、卫生、文化等社会事业投入，做强载体服务功能；加快凤翔县城新区开发，规划建设凤翔大道，加速与宝鸡主城区的对接；强力推进陇县南岸新城建设，加快中央商务区和东部商务区建设步伐。加快各具特色的县城建设，完善市政设施，提升服务功能，壮大特色产业，推动产城融合。抓好5个省级重点示范镇、4个文化旅游名镇（街区）和3个市级重点镇建设，加快建制镇规划编制和基础设施完善，年内实现10万农民进城落户。整合渭河综合治理、农村环境综合整治等项目资金，加快渭河两岸新型工业园区、现代农业示范园区、新型居民社区和生态景观示范区建设，推动“四区”融合发展，打造关中西部城镇群和渭河沿线统筹城乡示范带。

　　高水平精细化管理城市。紧紧围绕创建全国文明城市、国家生态园林城市，承办省十五运会、世界佛教徒联谊会四件大事，大处着眼，小处着手，注重细节，推进城市管理体制创新，完善管理制度，提高管理水平，努力塑造宝鸡城市新形象。下大力气改造建筑立面，规范门头牌匾，提高绿化水平，扮靓城市街景。规范和加强城市管理综合执法，大力整顿市区交通秩序，集中整治市区“摩的”、“黑出租”等非法营运行为，集中治理机动车乱停乱放、行人和非机动车闯红灯等交通违法行为，巩固渣土车专项整治成果，使城市干净整洁、文明有序，确保全国文明城市创建成功。深入实施“宽带普及提速”工程，推动智慧城市建设，提高数字化城管水平。推广渭滨区集贸市场数字化监管模式，规范市场经营秩序。建立物业投诉受理机制，保障业主合法权益。

　　科学构建交通网络。加快宝兰客专建设进度，开工建设宝汉高速宝鸡至坪坎段、连霍高速市区过境线潘家湾至苟家岭段，确保绛法高速建成通车，力争合凤高速提前建设。加快太白、陇县县城过境段、潘太公路、麟游到关中环线段、西宝中线陈仓段等干线公路改扩建，实施140公里特困地区交通扶贫重要县乡公路改造提升工程。做好宝鸡支线机场、关中城际铁路、宝鸡枢纽货运南环线、北绕城高速等重大交通项目前期工作。

　　六、持续优化生态环境，加快建设美丽宝鸡

全力推进城乡环境综合整治。实施村庄清洁行动，高标准、高质量完成225个村的省级连片整治示范项目，积极推进60个村的市级农村环境综合整治项目，巩固扩大整治成效。积极借鉴农村环境综合整治经验，在金台、渭滨、陈仓三区开展城中村、城郊村、居民小区、背街小巷环境卫生治理，使城市时尚典雅、农村干净整洁。大力开展西宝客专、连霍高速、宝平高速宝鸡段沿线环境综合整治。完成金河、绛帐、眉县霸王河工业园区污水处理厂建设，以及十里铺、陈仓区、高新区、麟游县、凤翔县污水处理厂提标改造工程，加快扶风县法门、绛帐垃圾无害化处理场、岐山县益店生活垃圾填埋场建设。

　　大力实施渭河流域综合整治。加快支流入渭口桥梁建设，实现渭河堤防全线贯通。有序推进渭河堤防硬化、绿化和滩区整治，完善提升渭河70公里绿色生态景观长廊。以重污染企业淘汰关停、重点污染源深度治理、河流生态修复为重点，实施渭河清水行动。完成市区一河两岸景观改造提升续建项目、代家湾段防洪暨生态治理、金陵河右岸堤顶硬化等建设任务。实施石坝河、瓦峪河市区段提升工程，整治周边环境。加强水资源保护，完善补偿机制。高标准推进清姜河、金陵河等市区18条河流整治，打造水景相望、生态优美、兼具健身休闲的“水润宝鸡”。

　　倾力做好大气污染防治。大气污染防治是重大民生问题，必须多管齐下，综合施治。继续开展“治污降霾•保卫蓝天”行动，以控煤、控车、控尘、控烟气为重点，拆除燃煤锅炉200台，开展居民用煤改气工作，削减燃煤总量；加强机动车尾气污染治理，淘汰黄标车、老旧机动车5000辆，划定不达标机动车禁行区域，斩断机动车“黑尾”；扩大市区主要行车道路机械清扫面积，健全房屋拆迁、建筑施工、道路扬尘等长效监管机制，对全市100个重点建筑工地进行综合整治；加大油气挥发污染治理。出台大气污染防治考核办法，建立联防联控机制，不断改善空气质量。

　　着力开展绿化美化亮化。继续实施绿色宝鸡九大工程，完成造林绿化45万亩，抓好西宝客专等交通沿线、秦岭北麓、渭河北坡绿色屏障建设，率先推进关中大地园林化，确保通过国家森林城市复查。积极创建国家生态园林城市、国家生态市，力争凤县创建国家级生态县通过考核验收，建成2个省级生态县和一批生态镇、生态村。加快千渭之会湿地公园建设，新建防护、景观林带8公里，种植湿地植物1500亩，打造市区生态“绿肺”。继续实施临街建筑物节能亮化工程，打造亮丽宝鸡。

　　七、切实保障改善民生，提高群众幸福指数

　　提升社会保障水平。深入推进国家创业型城市创建，积极开展鼓励创业活动，新增小额担保贷款10亿元。拓宽就业领域，年内实现城镇新增就业5万人，农村劳动力转移100万人以上。力争实行城乡居民养老保险市级统筹，提升社会保障体系管理水平。加快绛帐社会福利院整体迁建、市社会福利院老年公寓楼二期项目建设，推广渭滨区居家养老、千阳县农村互助养老模式，大力开展“星级敬老院”创建活动，完善以居家为基础、社区为依托、机构为支撑的社会养老服务体系。健全农村“三留守”人员关爱服务体系。强力推进城镇棚户区改造步伐，加快龙山雅居、虢镇名苑、蟠龙新居保障性住房建设，新开工3.6万套，其中棚户区改造不少于2万套。建立保障房动态管理机制，做到公平分配。加快农村“三无户”安全住房工程建设步伐，年内完成1000户建房任务。

　　优先发展教育事业。启动教育现代化先进县区创建，普及优质教育资源，实现均衡发展。以增加数量、提高质量为着力点，加强市区和县城义务教育学校建设，有效破解择校热、大班额问题。落实新一轮学前教育三年行动计划，抓好公办幼儿园建设，切实规范办园行为。推进民办教育发展，提升办学水平。加强职业教育，培养高素质劳动者和技能型人才。推动宝鸡职业技术学院建成省级示范性院校。支持宝鸡文理学院转型发展，建设工科突出的多科性大学。

　　提高医疗服务能力。加快市精神卫生中心整体搬迁、市儿童医院改扩建等项目建设进度。深入实施乡镇卫生院安心工程和基层中医药服务能力提升工程，加强乡村医生队伍建设，让群众就近享受优质医疗服务。完善大病保险、慢性病救助制度，解决群众因病致贫、因病返贫问题。启动健康城市创建工作，继续开展卫生应急综合示范县（区）和慢性病综合防控示范区创建活动。真切关心失独家庭，认真落实计划生育新政策，提高服务对象的健康保障水平。

　　发展文化体育事业。加快市文化艺术中心前期工作，力争年内开工建设。实施基层文化场馆提升和文化器材配送工程，不断满足群众文化需求。持续开展专项治理，维护全市文化市场和网络文化秩序。启动实施北首岭、九成宫等遗址保护规划，力争雍城遗址保护规划通过省政府颁布，周原遗址保护规划通过省上论证。办好中国两岸四地道文化论坛。按期完成游泳跳水馆、射击射箭馆建设和市体育场馆改造任务，做好接待服务和环境保障工作, 全力筹办好省十五运会。完善城乡体育设施，加强组织引导，推动群众体育活动蓬勃开展。

　　关爱三大困难群体。全面实施六盘山片区和秦巴山片区区域发展与扶贫攻坚规划，持续实施突破西山战略，抓好整村推进等“六大扶贫工程”，完成移民搬迁2000户，实现12万农村贫困人口脱贫。完善动态管理机制，提高城乡低保科学化、精细化管理水平，做到应保尽保。积极探索政府购买服务，实施残疾人居家托养救助模式，切实抓好“双千助残工程”，为残疾人办好“十件实事”。加大社会救助力度，努力提高困难群众的生活水平。

　　推进社会治理创新。完善社区网格化管理机制，健全“一楼一册、一户一档”管理体系。放手发展各类社会组织。全面完成第三次经济普查工作任务。深化社会治安立体化防控体系建设，持续开展严打整治，打造最安全城市。深入实施“六五”普法规划，加快推进法治宝鸡建设。积极开展“百镇（街）千村(社区)创双无、化解矛盾促和谐”行动，着力解决信访突出问题。全面落实维护社会稳定风险评估机制,从源头上预防和减少社会不稳定因素。深入开展食品药品安全示范县区创建活动。实施从田间到餐桌、从生产到流通全过程的监督，确保舌尖上的安全。严格落实安全生产监管责任和企业主体责任，切实抓好消防安全、交通安全和校园安全，有效预防各类安全事故发生，建设“平安、法治、和谐”宝鸡。

　　八、全面深化各项改革，努力增强发展活力

　　按照中省统一部署，从最紧迫的事项、老百姓最期盼的领域、最突出的问题、最易达成共识的环节推动改革，促进经济社会更有效率、更加公平、更可持续发展。

加快行政体制改革。严格控制机构编制，基本完成市县政府机构改革，推进事业单位分类改革。下决心再取消和下放一批行政审批事项，优化审批程序，让企业和群众少跑路、少受累。创新市场监管方式，推动工商注册制度便利化，统筹准入管理与事中、事后管理，对妨碍统一市场和公平竞争的各种规定进行清理，探索建立城乡统一的建设用地市场。积极推进城市管理体制改革，使管理重心下移。创新体制机制，促进各类园区科学发展。

　　扎实做好国企改革。加快秦岭化肥总厂分块改制步伐，稳步实施北照公司、五一纸厂、第一染织厂等3户企业政策性破产，完成省电子信息集团重组陕铜公司，引进行业优势企业参与国企改革。完善国有资产管理体制，探索由管企业为主向管资本为主转变，逐步建立分类监管模式。积极发展混合所有制经济，支持国有企业与民营、集体、外资等各类企业融合发展。

　　加大财税金融改革。扩大“营改增”范围，做好资源税等税种改革准备工作。全面公开部门预算，确保财政资金支付规范有效、公开透明。继续深化财政专项资金管理改革，提高资金使用效益。抓好科技与金融结合试点，实施“四对接一扩大”金融服务提质增效工程，大力发展普惠金融。加快城市建设投融资体制改革，理顺城投体制，吸引民间资本参股市县城投公司。

　　全面深化农村改革。积极探索农村集体所有制的有效实现形式，落实集体所有权，稳定农户承包权，放活土地经营权，加快构建以农户家庭经营为基础、合作与联合为纽带、社会化服务为支撑的现代农业经营体系。发展多种形式的规模经营，促进土地向种植大户、家庭农场、专业合作社集中。

　　推进社会事业改革。加快文化体制改革，对已经转企改制的国有文化企业，建立完善现代企业制度，对公益性文化事业单位，深化内部体制改革，推动政府部门由办文化向管文化转变。推进教育综合改革，完善普通高中招生制度，开展中小学教育质量评价试点。深化医药卫生体制改革，以六大领域为重点，扎实开展卫生综合改革试点；探索建立医院法人治理结构，完善人事分配制度，激发发展活力；改革医保支付方式，提升医疗保障水平。探索建立城乡居民丧葬补助金制度，积极推进殡葬改革，倡导文明丧葬新风。

　　切实加强政府自身建设

　　打铁还需自身硬。面对艰巨繁重的改革发展任务，我们将牢记宗旨，狠刹“四风”，勤勉尽责，永不懈怠，努力建设为民、务实、清廉、高效的服务型政府。

倾情亲民为民。扎实开展党的群众路线教育实践活动，强化纪律建设，持之以恒纠正“四风”问题，提高服务基层的能力和水平。切实转变政府职能，完善服务承诺、首问负责、限时办结等制度，优化环境，提升效能。进一步扩大政府信息公开，完善政府门户网站，推进电子政务建设。组织开展邀请公民代表进政府和万名群众评百名科长活动，深入推行“百姓问政”，让监督的阳光照亮权力运行的每一道环节、每一个角落。畅通群众利益诉求表达渠道，关心群众冷暖，体察民生艰难，真心实意为民办实事、办好事，以干部的“辛苦指数”提升人民群众的幸福指数。

　　用心谋事干事。强化责任意识，真正把精力放在抓落实上，把心思放在干事创业上，把本领用在促进发展上。要有“功成不必在我”的境界，不贪一时之功，不图一时之名，甘做铺垫之事，甘抓未成之事。建立健全责任体系，做到事有专管之人、人有明确之责、责有限定之期。大兴求真务实之风，抓铁有痕，踏石留印，市政府抓的事情，一定要议立决、决立行，不争论、不折腾，脚踏实地地干，实事求是地做，不见成效不收兵。完善考核制度，加强效能监察，强化督办问效，加大行政问责，切实解决不作为、慢作为问题，确保政令畅通，用抓发展的实效取信于民。

　　坚持依法守法。大兴调查研究之风，深入基层，倾听群众的意见和呼声，使各项决策真正代表人民的利益。完善和落实重大决策专家咨询、社会公示、社会听证和责任追究制度，加强对重大政策实施的绩效评估。自觉接受人大、政协的监督，虚心听取社会各界的意见、建议，认真办理人大议案、代表建议和政协委员提案，努力提高政府工作水平。严格落实行政执法责任制和执法过错追究制，保证政府的各项工作依法进行。

　　坚守清正清白。认真落实中央八项规定，坚决执行《党政机关厉行节约反对浪费条例》、《党政机关国内公务接待管理规定》等规章制度，进一步压缩“三公”经费，降低行政成本，把有限的资金用在经济发展和改善民生上，政府自觉过“紧日子”，让群众过上“好日子”。严格落实党风廉政建设责任制，执行好廉洁从政各项规定，坚持用制度管权、管事、管人，把权力关进制度的笼子里，让干部学会在约束中工作、习惯在监督下干事。健全建筑工程、土地交易、政府采购等重点领域的反腐倡廉长效机制，强化对财政资金、重大投资项目的审计监督。坚持以零容忍的态度反对腐败，抓早抓小，坚决查处违法违纪案件，建设廉洁政府,做到干部清正、政府清廉、政治清明。

　　各位代表，回顾过去，我们满怀豪情；展望未来，我们充满信心。让我们倍加珍惜来之不易的发展势头，倍加珍惜深化改革的难得机遇，倍加珍惜团结和谐的良好局面，在市委的坚强领导下，坚定信心，振奋精神，锐意进取，扎实工作，为开创宝鸡更加美好的明天而努力奋斗！
